# Hereditary Transthyretin Amyloidosis in Brazilian Amazon Caused by a Globally Rare Variant Predominant in Asia

**DOI:** 10.1016/j.jaccas.2026.109924

**Published:** 2026-09-23

**Authors:** Matheus Martins Monteiro, Marcela Oliveira de Azevedo, Rodrigo Fernandes de Castro, Moises Abtibol Machado, Fernando Almeida Bezerra, Nayara do Carmo Santos Lima, Diogo Corrêa Lamberti, Andreza Araújo de Oliveira, Kátia do Nascimento Couceiro, João Marcos Bemfica Barbosa Ferreira

**Affiliations:** a Hospital Beneficência Portuguesa de São Paulo, São Paulo, São Paulo, Brazil; b Escola Superior de Ciências da Saúde, Universidade do Estado do Amazonas (UEA), Manaus, Amazonas, Brazil; c Faculdade de Medicina, Universidade Nilton Lins, Manaus, Amazonas, Brazil

**Keywords:** cardiomyopathy, chronic heart failure, genetic disorders

## Abstract

**Background:**

Hereditary transthyretin amyloidosis (ATTRv) exhibits marked phenotypic heterogeneity. The p.Asp58Ala variant is globally rare and has not been reported in major Brazilian epidemiologic studies.

**Case Summary:**

We report 10 patients with ATTRv-p.Asp58Ala, including 6 index cases and 4 identified through family screening. Mean left ventricular ejection fraction was 64.7%, with a mean interventricular wall thickness of 14.1 mm and a mean posterior wall thickness of 12.9 mm. Mixed cardiac and neurologic involvement predominated (60%), followed by asymptomatic (30%) and isolated cardiac (10%) phenotypes. Among 7 patients under follow-up, 2 receive tafamidis and 1 receives inotersen.

**Discussion:**

Similar to Korean and other Asian cohorts, mixed phenotypes predominated, although our series showed substantial cardiac involvement and significant mortality.

**Take-Home Messages:**

p.Asp58Ala is a rare ATTR variant, previously described mainly in Asian populations. Its identification in the Amazon region suggests a possible previously unreported regional clustering in Brazil.

## Visual Summary

**Figure:**
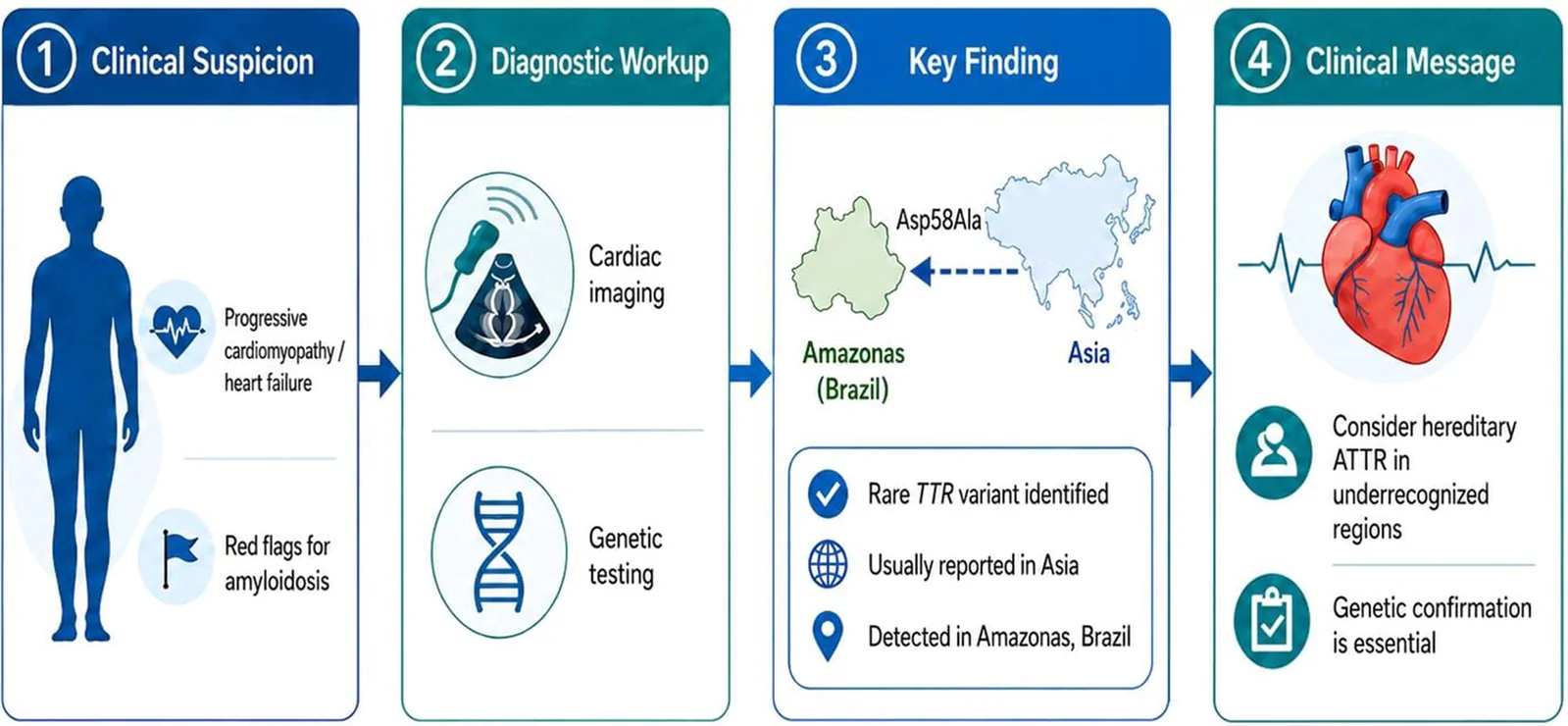

Amyloidosis comprises a group of diseases characterized by the extracellular deposition of misfolded proteins that accumulate in organs and tissues, leading to progressive dysfunction. When cardiac involvement occurs, it is defined as cardiac amyloidosis, a condition associated with significant prognostic impact. Among the various precursors described, transthyretin (TTR) and immunoglobulin light chains account for the majority of cases of amyloid cardiomyopathy.^1^^,^^2^

In hereditary TTR amyloidosis (ATTRv), more than 140 variants of the TTR gene have been described, with heterogeneous clinical expression. Among the most common variants in Brazil are Val50Met and Val142Ile. The Asp58Ala variant is considered globally rare and has not been described in major epidemiologic studies conducted in Brazil. In Asian populations, particularly in South Korea, it has been reported as the most common variant. Although p.Val50Met is frequently associated with neurologic manifestations and p.Val142Ile with a predominantly cardiac phenotype, rare variants such as p.Asp58Ala present limited geographic distribution and remain poorly characterized, expanding the genetic and clinical spectrum of the disease.^3^^,^^4^

## Case Series

### History of presentation

This descriptive case series was conducted at a cardiology referral center in the Amazon region. Patients with hereditary transthyretin amyloidosis (ATTRv) carrying the p.Asp58Ala variant confirmed by genetic testing were included. In recent years, 10 patients with ATTRv associated with the p.Asp58Ala variant were diagnosed at our center, including 6 index cases and 4 identified through family screening. Within the local cohort of hereditary amyloidosis, this variant accounted for 27.3% of index cases, a proportion equivalent to that observed for the Val50Met variant during the same period, making it one of the most frequent mutations in our sample (unpublished data). The mean age at diagnosis was 55.8 ± 13.9 years, with a predominance of female patients (80%) (Table 1). A marked geographic clustering of cases was observed, with 70% of patients originating from the municipality of Maués, Amazonas, followed by Manaus (20%) and Barcelos (10%), suggesting possible regional aggregation of this mutation.

**Table 1:** Clinical and Phenotypic Characteristics of Patients With Hereditary Transthyretin Amyloidosis (ATTRv) Associated With the p.Asp58Ala Variant

Patient	Case	Sex	Age, y	Phenotype	Diagnosis	Extracardiac Manifestations
1	Index	M	67	Mixed	PYP scan	Sensory changes; weight loss
2	Family	F	41	Asymptomatic	Genetic testing	None
3	Family	F	48	Asymptomatic	Genetic testing	None
4	Index	F	71	Mixed	Genetic testing	Dysautonomia
5	Index	F	70	Mixed	Genetic testing	Sensory changes
6	Index	F	66	Mixed	Genetic testing	Sensory changes; weight loss
7	Family	F	32	Asymptomatic	Genetic testing	None
8	Family	F	40	Mixed	Genetic testing	Sensory changes
9	Index	F	62	Mixed	PYP scan	Sensory changes; dysautonomia; weight loss
10	Index	M	61	Cardiac	Genetic testing	None

PYP = pyrophosphate scintigraphy.

### Past medical history

Relevant past medical history was not systematically available for all patients in this case series.

### Differential diagnosis

Genetic testing was performed to confirm the p.Asp58Ala variant and to evaluate other causes of hypertrophic cardiomyopathy and infiltrative cardiac diseases, including sarcomeric hypertrophic cardiomyopathy.

### Investigations

Clinical, demographic, phenotypic, and echocardiographic data were obtained through systematic medical record review, including analysis of cardiac structural characteristics, clinical presentation, and disease progression during follow-up. Regarding clinical presentation, the mixed phenotype (cardiac and neurologic involvement) was the most prevalent (60%), followed by asymptomatic cases (30%) and isolated cardiac involvement (10%), demonstrating significant phenotypic heterogeneity (Table 1). Echocardiographic parameters were unavailable for 1 asymptomatic patient because transthoracic echocardiography was not performed because of personal reasons. Among the other 9 cases, the mean left ventricular ejection fraction was 64.7% ± 10.3%, with a mean interventricular wall thickness of 14.1 ± 5.2 mm and a mean posterior wall thickness of 12.9 ± 3.7 mm, findings consistent with structural cardiac involvement (Table 2). NT-proBNP and troponin levels were available for only a minority of patients and therefore were not systematically included in the table to avoid introducing incomplete data.

**Table 2:** Echocardiographic Features and Clinical Outcomes of Patients With Hereditary Transthyretin Amyloidosis (ATTRv) Associated With the p.Asp58Ala Variant

Patient	LVEF (%)	IVS Thickness (mm)	PW Thickness (mm)	Follow-Up (mo)	Status
1	55	22	14	9	Follow-up
2	NR	NR	NR	8	Follow-up
3	74	8	9	12	Follow-up
4	65	16	15	18	Death
5	60	13	13	8	Follow-up
6	75	19	17	12	Follow-up
7	72	9	9	12	Follow-up
8	77	7	7	18	Follow-up
9	54	16	17	3	Death
10	50	17	15	8	Death

IVS = interventricular septum; LVEF = left ventricular ejection fraction; NR = not reported; PW = posterior wall.

### Management (medical/interventions)

Among the 7 patients under follow-up, 2 with a mixed phenotype are receiving tafamidis, a selective TTR stabilizer, whereas another is being treated with inotersen, a disease-modifying therapy approved for ATTRv with early-stage polyneuropathy. The 4 relatives identified through family screening remain under specialized follow-up, allowing early monitoring and the possibility of timely therapeutic intervention.

### Outcome and follow-up

During follow-up, 3 of the 6 index cases died (50%), with 2 deaths occurring within the first year and 1 after 18 months (Table 2), underscoring the potential severity of the disease. These patients were receiving tafamidis 20 mg/d, as the recommended 80 mg/d dose was not available through the Brazilian public health system during the study period.

### Discussion

**Figure 1:**
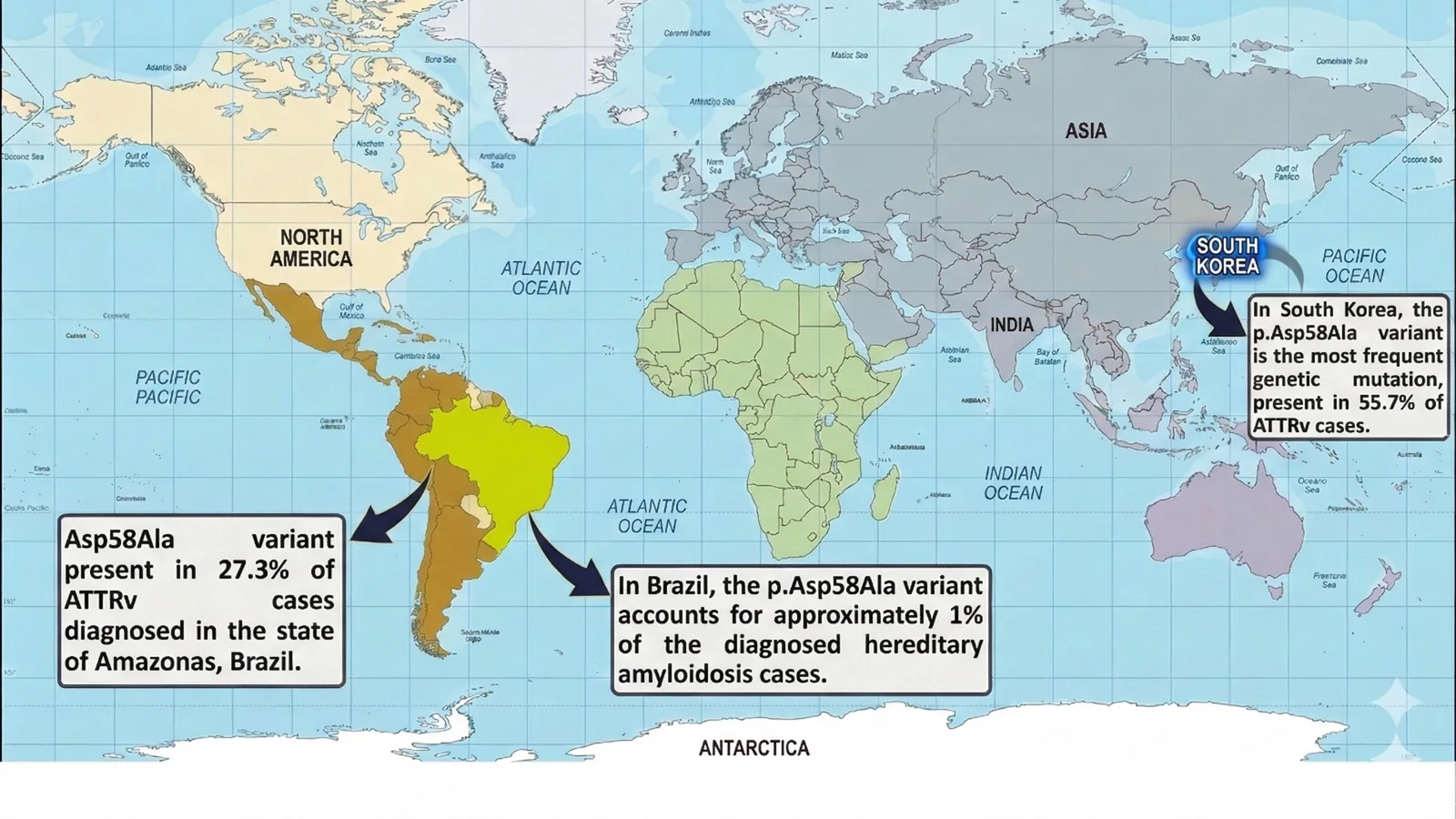
Clinical Characteristics and Geographic Distribution of Hereditary Transthyretin Amyloidosis Associated With the Rare p.Asp58Ala Variant in Patients From the Brazilian Amazon ATTRv = hereditary transthyretin amyloidosis.

The p.Asp58Ala variant of the TTR gene is considered rare in the global context of ATTRv. However, Jang et al,^5^ when describing a Korean cohort, identified p.Asp58Ala as the most frequent mutation in that specific study group, predominantly associated with a mixed phenotype and later disease onset. This finding demonstrates that, although globally rare, the distribution of TTR variants may present distinct regional patterns, possibly related to geographic clustering or founder effects, as illustrated in Figure 1.

Another study conducted in South Korea, including 105 patients with TTR amyloid cardiomyopathy, reported the Asp38Ala variant (corresponding to Asp58Ala) as the most frequent genetic mutation, representing 55.7% of variant cases. In that cohort, neurologic manifestations were common and, in some patients, the absence of significant ventricular hypertrophy at diagnosis was observed.^6^ Although this variant is considered globally rare and mainly described in Asian populations, our findings demonstrate its presence in the Amazon region as well.

In addition to the Asp58Ala substitution, other alterations involving the same residue, such as p.Asp58Val and p.Asp58Tyr, have been reported in recent cases of amyloidosis, reinforcing that codon 58 represents a relevant position in the TTR protein. The recurrence of different substitutions at this residue suggests a possible functional impact on the stability of the TTR tetramer, favoring destabilization and amyloid deposition.^7^^,^^8^ In this context, the identification of this case series of p.Asp58Ala in the Amazon region expands its clinical relevance and potential impact.

The literature on ATTRv emphasizes that the phenotypic expression of TTR variants is highly heterogeneous, ranging from isolated neuropathy to predominant cardiac involvement or mixed forms, as described in broad reviews of the clinical spectrum of the disease.^9^ In our series, a predominance of the mixed phenotype was observed, similar to that reported in the Asian cohort, but with a relevant proportion of structural cardiac involvement and significant mortality among index cases. These data suggest possible variations in penetrance and clinical severity when compared with other described populations.^5^

The Brazilian Society of Cardiology Position Statement (2021) highlights the broad genetic heterogeneity of ATTRv, with more than 140 variants described in the TTR gene and variable clinical expressivity. Although p.Val50Met and p.Val142Ile are more prevalent in Brazil, rare variants must also be considered, and molecular diagnosis is essential for etiologic confirmation and family screening. In this context, the recurrent identification of p.Asp58Ala in the Amazon region expands the national genetic spectrum and suggests a possible regional concentration with epidemiologic relevance.^10^

## Conclusions

The identification of multiple cases of ATTRv associated with the Asp58Ala variant in the Amazon region expands the genetic and geographic spectrum of ATTRv in Brazil. Although this variant is considered globally rare and more frequently described in Asian populations, our findings highlight its presence in the Brazilian Amazon. These results reinforce the importance of molecular diagnosis and family screening in populations from this region, contributing to early recognition, improved prognostic stratification, and a broader understanding of the genetic diversity of the disease

**Take-Home Messages**

• p.Asp58Ala is a rare transthyretin amyloidosis variant, previously described mainly in Asian populations.
• Its identification in the Amazon region suggests a possible previously unreported regional clustering in Brazil..

**Visual Summary:**
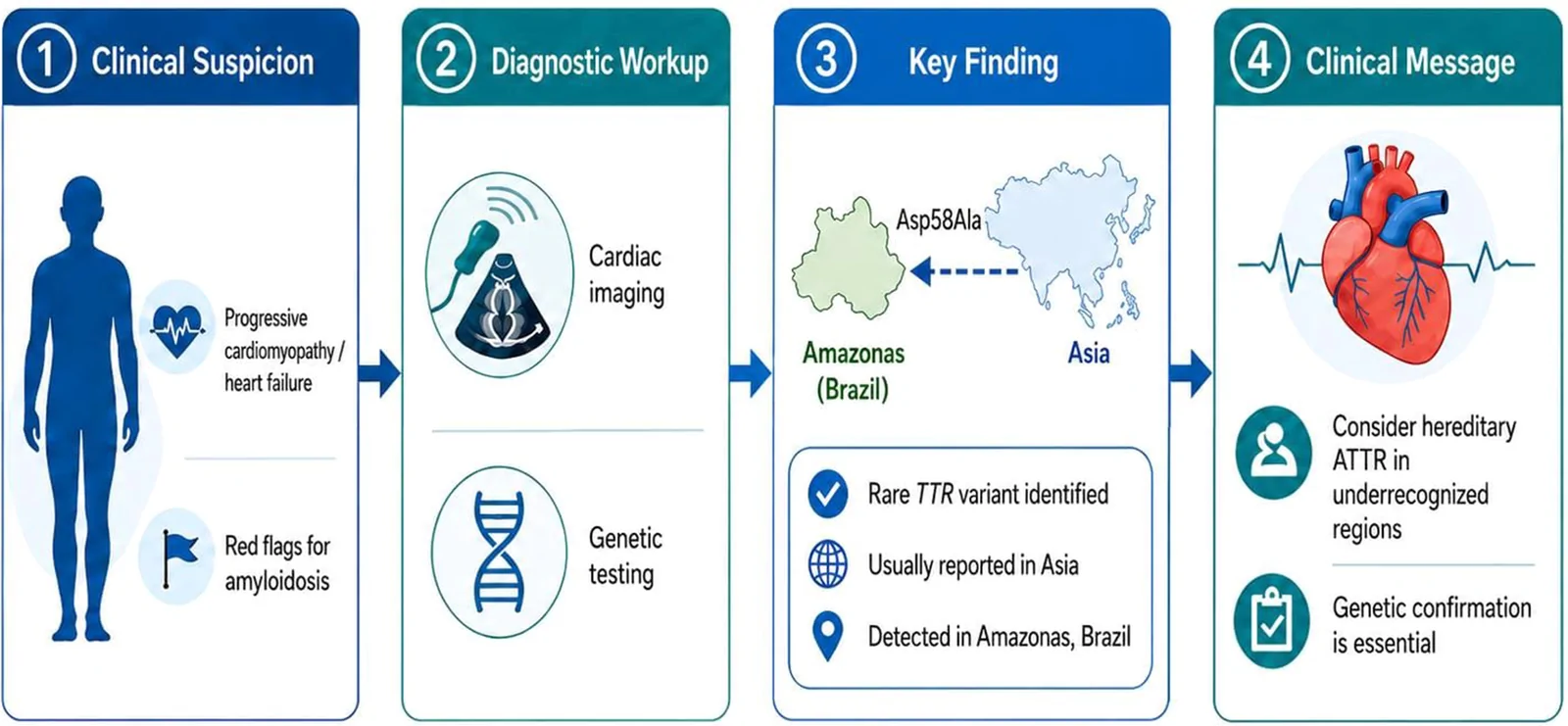
Hereditary Transthyretin Amyloidosis in the Brazilian Amazon The visual summary illustrates the clinical suspicion, diagnostic investigation, and identification of a rare pathogenic transthyretin (TTR) variant predominantly reported in Asia in a patient from Amazonas, Brazil. The dashed arrow represents the geographic association from Asia to Amazonas.

## Funding Support and Author Disclosures

The authors have reported that they have no relationships relevant to the contents of this paper to disclose.
